# New insights into mitral heart valve prolapse after chordae rupture through fluid–structure interaction computational modeling

**DOI:** 10.1038/s41598-018-35555-5

**Published:** 2018-11-23

**Authors:** Andrés Caballero, Wenbin Mao, Raymond McKay, Charles Primiano, Sabet Hashim, Wei Sun

**Affiliations:** 1 The Wallace H. Coulter Department of Biomedical Engineering, Georgia Institute of Technology and Emory University, Atlanta, GA USA; 2 Cardiology and Cardiac Surgery, The Hartford Hospital, Hartford, Connecticut USA

**Keywords:** Valvular disease, Translational research, Biomedical engineering

## Abstract

Mitral valve (MV) dynamics depends on a force balance across the mitral leaflets, the chordae tendineae, the mitral annulus, the papillary muscles and the adjacent ventricular wall. Chordae rupture disrupts the link between the MV and the left ventricle (LV), causing mitral regurgitation (MR), the most common valvular disease. In this study, a fluid-structure interaction (FSI) modeling framework is implemented to investigate the impact of chordae rupture on the left heart (LH) dynamics and severity of MR. A control and seven chordae rupture LH models were developed to simulate a pathological process in which minimal chordae rupture precedes more extensive chordae rupture. Different non-eccentric and eccentric regurgitant jets were identified during systole. Cardiac efficiency was evaluated by the ratio of external stroke work. MV structural results showed that basal/strut chordae were the major load-bearing chordae. An increased number of ruptured chordae resulted in reduced basal/strut tension, but increased marginal/intermediate load. Chordae rupture in a specific scallop did not necessarily involve an increase in the stress of the entire prolapsed leaflet. This work represents a further step towards patient-specific modeling of pathological LH dynamics, and has the potential to improve our understanding of the biomechanical mechanisms and treatment of primary MR.

## Introduction

MR is the most common valvular heart disease, with a prevalence of 9.3% in the US population aged 75 and above^1^. MV prolapse following rupture of the chordae tendineae is a major cause of primary MR, and is associated with several causes such as bacterial endocarditis, local myxomatous degeneration, connective tissue abnormalities, hypertrophic cardiomyopathy, and blunt chest trauma^2^. Mild varieties of mitral chordae rupture generally involve separation of a single chorda, which can lead to minimum hemodynamic effects and requires neither intervention nor treatment. Rupture of multiple chordae, however, may cause moderate to severe acute or chronic MR^2^. Studies have shown that chordae rupture may be either immediate onset or a progressive process in which minimal rupture precedes more extensive or even complete rupture^3^. To restore normal MV function and prevent further pathologic progression, MV repair or replacement can be performed^2^. A variety of surgical and transcatheter repair treatments are currently available, however, in many cases there is a sub-optimal selection of the treatment attributed to the complicated repair techniques and the mitral apparatus complex geometry that require extensive surgical experience and specialized skills^4^.

Computational modeling of heart valve dynamics and function is an active research field that not only can allow an in–depth examination of the dynamics of the LV-valve complex under normal and diseased states, but can also offer potential to inform the therapeutic decision-making process^5^. Finite element (FE) studies have been performed in the past with the goal to investigate MV dynamics after chordae rupture. Kunzelman and colleagues^6,7^ were among the first to study the effects of chordae rupture and their repair on MV function. More recently, Kim *et al*.^8–10^ and Sturla *et al*.^11,12^ evaluated the biomechanical characteristics of MV models with posterior mitral leaflet (PML) prolapse, and compared different repair techniques such as neo-chordae and leaflet resection. In these FE studies, chordae structure was mainly determined from published clinical data and *ex vivo* findings due to the limited resolution of the medical image data. Moreover, FE or structural-only models assume the hemodynamic pressure load is uniformly distributed over the leaflet surface, a factor which has been previously shown to affect valve dynamics^13,14^. Structural valve models are appropriate for simulation of quasi-static events such as closed valves, but in order to accurately model full dynamic/transient valve dynamics, an FSI modeling approach that accounts for the strong coupling between the large deformation of the leaflets and the intraventricular blood flow is required^13^.

The first FSI model to investigate MV function with ruptured chordae was presented by Toma *et al*.^15^. In this study, the importance of individual chordae on MV closure dynamics was assessed by simulating rupture in every of the 51 possible chordae branches. However, a rigid tube was used as an approximation of the LV domain, neglecting the cardiac wall motion and its direct effect on the blood flow. The severity of the resultant MR was also approximated by calculating the regurgitant orifice area, with no hemodynamic variables analyzed. In the study by Khodaei *et al*.^16^, although the modeling approach included the LV geometry to account for a physiologic moving boundary, several critical simplifications such as an idealized MV geometry, the omission of the LA and aortic valve (AV) geometries, and the modeling of only the valve closing phase limited the findings of the study. Due to the complex mechanical coupling between the MV and the LV mediated through the papillary muscles (PM), the chordae tendineae and the dynamic mitral annulus (MA), rigorous modeling of MV dynamics under normal and diseased states should include the entire LH complex. Indeed, it has been determined that even with a rigid U-shaped LV model, the intraventricular mitral flow patterns are substantially different from that estimated using a tubular geometry^13^.

In the present study, we utilized a comprehensive 3D LH model previously developed^17,18^ to investigate the coupled LV-valve pathological dynamics following mitral chordae rupture. The FSI framework used in this study takes into account the complexity of the LH morphology, the large deformation experienced by the aortic and mitral leaflets and the cardiac wall, the anisotropic nonlinear elastic behavior of the valvular tissue, and the pulsatile hemodynamic loads during the entire cardiac cycle. FSI simulations of LH dynamics under physiologic (control) and seven chordae rupture conditions are performed and compared, and the effects on the force balance of the mitral apparatus, leaflets deformation state, MR severity, LH hemodynamics and overall cardiac efficiency are investigated.

## Results

### Global hemodynamic parameters

**Figure 1:**
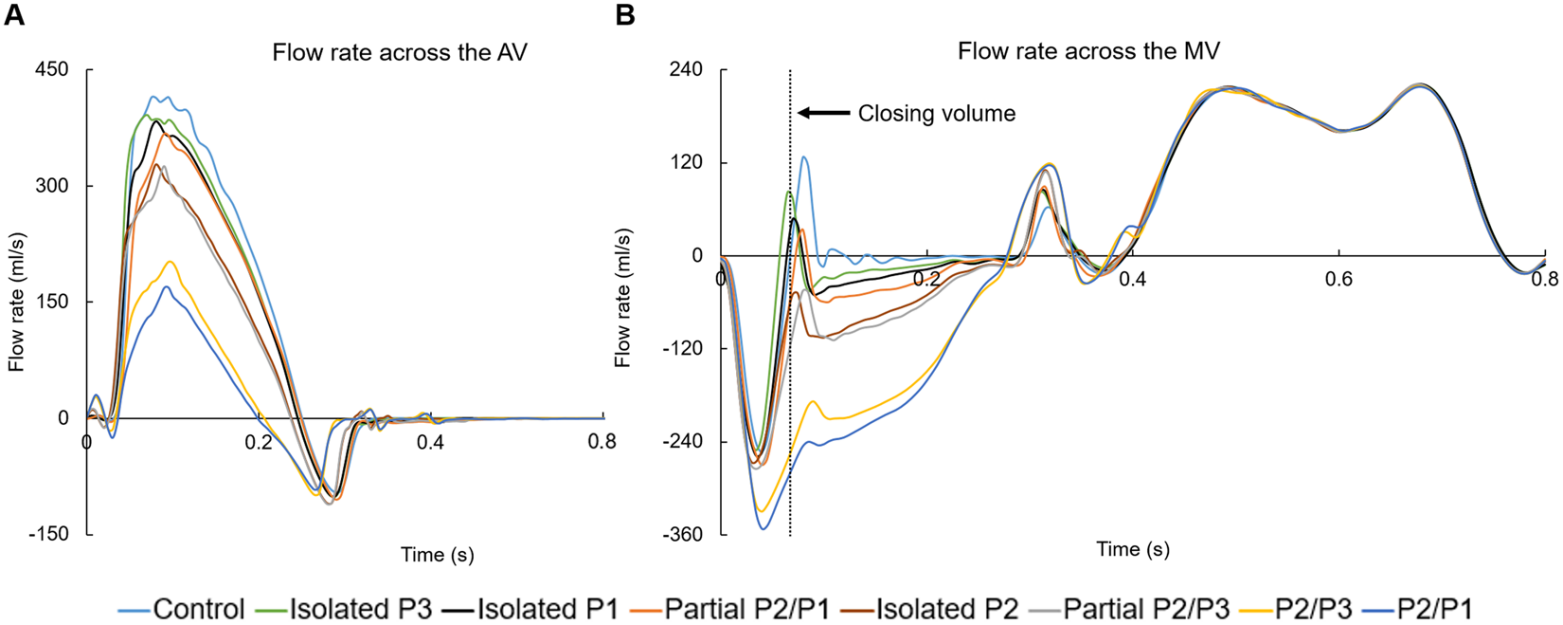
Flow rate across the AV and MV through the cardiac cycle. PML is divided into P1, P2 and P3 scallops.

Figure 1 shows the computed volumetric flow rate through the AV and MV from systole to diastole for the LH models. The negative AV flow at early diastole in Fig. 1A indicates backflow of blood into the LV during AV closure, while the negative systolic MV flow in Fig. 1B indicates the backflow of blood into the LA due to MV closing and incompetence. During systole, the AV flow reached a peak value of 414 ml/s for the control model, while the minimum peak flow was obtained for the P2/P1 model, with a value of 169 ml/s. As seen in Fig. 1B, this reduction in the aortic flow from the P2/P1 model coincided with the highest mitral regurgitant flow.

In order to quantitatively grade the severity of MR for the different LH models, the MV regurgitant volume (*RV*_*MV*_) was determined by the time integral of the negative transmitral systolic flow, where *RV*_*MV*_ is equal to the sum of the closing volume and the leakage volume. In this study, the closing volume was defined as the volume of blood flowing retrograde through the valve during its closure, and was quantified for all models from the start of negative transmitral flow at early systole until the control model reached a positive mitral flow, as indicated by the dotted line in Fig. 1B. Any fluid volume accumulation after valve closure resulted from leakage and it was referred to as leakage volume. We note that for MR cases, there is no widely-accepted definition to distinguish between the closing and leakage volumes. The combination of the forward stroke volume (*SV*_*AV*_) and the *RV*_*MV*_ is known as the total SV of the LV (*LVSV*), which was used to calculate the regurgitant fraction, *RF*_*MV*_ = *RV*_*MV*_/*LVSV*. Results are summarized in Table 1.

**Table 1:** Summary of global hemodynamic parameters.

	Control	Isolated P3	Isolated P1	Partial P2/P1	Isolated P2	Partial P2/P3	P2/P3	P2/P1
RV_AV_ (ml)	4.27	4.34	4.63	4.73	4.61	5.05	4.62	4.72
MV closing volume (ml)	8.65	8.54	10.21	11.26	11.86	13.19	16.25	17.08
MV leakage volume (ml)	0.62	3.36	4.54	6.47	12.46	13.33	31.04	34.45
RV_MV_ (ml)	9.27	11.90	14.75	17.73	24.32	26.52	47.19	51.53
SV_AV_ (ml)	58.22	55.18	51.55	48.43	41.94	39.72	20.46	16.16
RF_MV_ (%)	13.74	17.74	22.25	26.80	36.70	40.04	69.76	76.13
MR severity (RF_MV_)	Mild	Mild	Mild	Mild	Moderate	Moderate	Severe	Severe

By using the *RF*_*MV*_ as a the parameter in the grading of MR severity^19^, Table 1 shows that from the seven chordae rupture models, 3 can be classified as having mild MR, 2 moderate MR, and 2 severe MR. As per 2014 AHA/ACC guidelines^20^, 2 chordae rupture models can be classified as having severe MR (*RF*_*MV*_ > 50%), while the other 4 models as having progressive MR (<50%). As expected, regurgitant volume in the AV, *RV*_*AV*_, was similar for all LH models, with the lowest value obtained for the control model. From these results it can be seen that the severity of MR not only has an impact on the *RV*_*MV*_ but also on the onset of AV closing (especially in the severe MR models) and on the *RV*_*AV*_ due to coupled valve dynamics.

### Chordae tension

**Figure 2:**
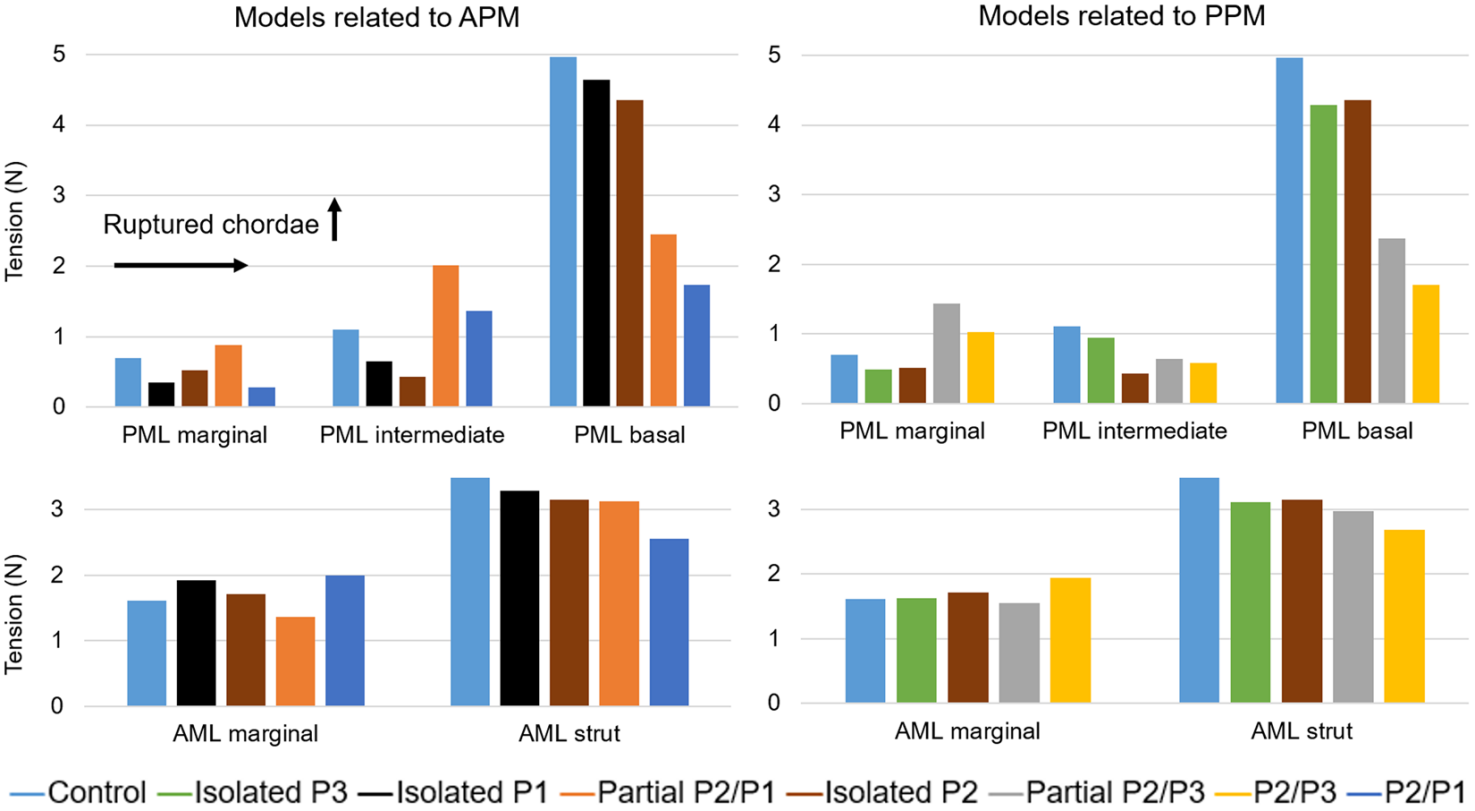
Chordae tension at peak systole. APM: anterolateral PM, PPM: posteromedial PM.

Figure 2 shows the net force carried by the different chordae tendineae groups at peak systolic pressure. The force experienced by a particular chordae group was calculated as the sum of vectors representing the tension in each individual chorda attached to that chordae group. In order to facilitate visualization and analysis, LH models were grouped according to the PM from where the chordae ruptured. Supplementary Table 1 presents the numerical values of Fig. 2.

In the control model, the highest PML chordae tension was found for the basal chordae, followed by the intermediate chordae, and lastly the marginal chordae. For the AML, the chordae tension was higher in the strut chordae than in the marginal chordae. When compared to the control model, Fig. 2 shows that there was no evident change in the tension of AML marginal chordae as the number of ruptured chordae increased. However, there was a noticeable increase in their tension (>20%) during total double scallop prolapse (i.e. P2/P3 and P2/P1 models). On the other hand, the tension in the strut chordae appeared to decrease as the number of ruptured chordae increased. This reduction was more than 20% during total double scallop prolapse.

In general, the tension in the PML basal chordae decreased as the as the number of ruptured chordae increased. Moreover, there was a clear reduction of more than 50% in their tension when partial or total double scallop prolapse occurred. In the isolated scallop prolapse models, as expected, the net PML marginal and intermediate chordae tensions decreased. However, in the partial double scallop prolapse models, marginal chordae tension increased more than 20% compared to the control model. When total double scallop prolapse occurred, while marginal chordae tension decreased and intermediate chordae tension increased in the P2/P1 model, the opposite trend occurred in the P2/P3 model, where marginal chordae tension increased and intermediate chordae tension decreased.

### Leaflet stress

**Figure 3:**
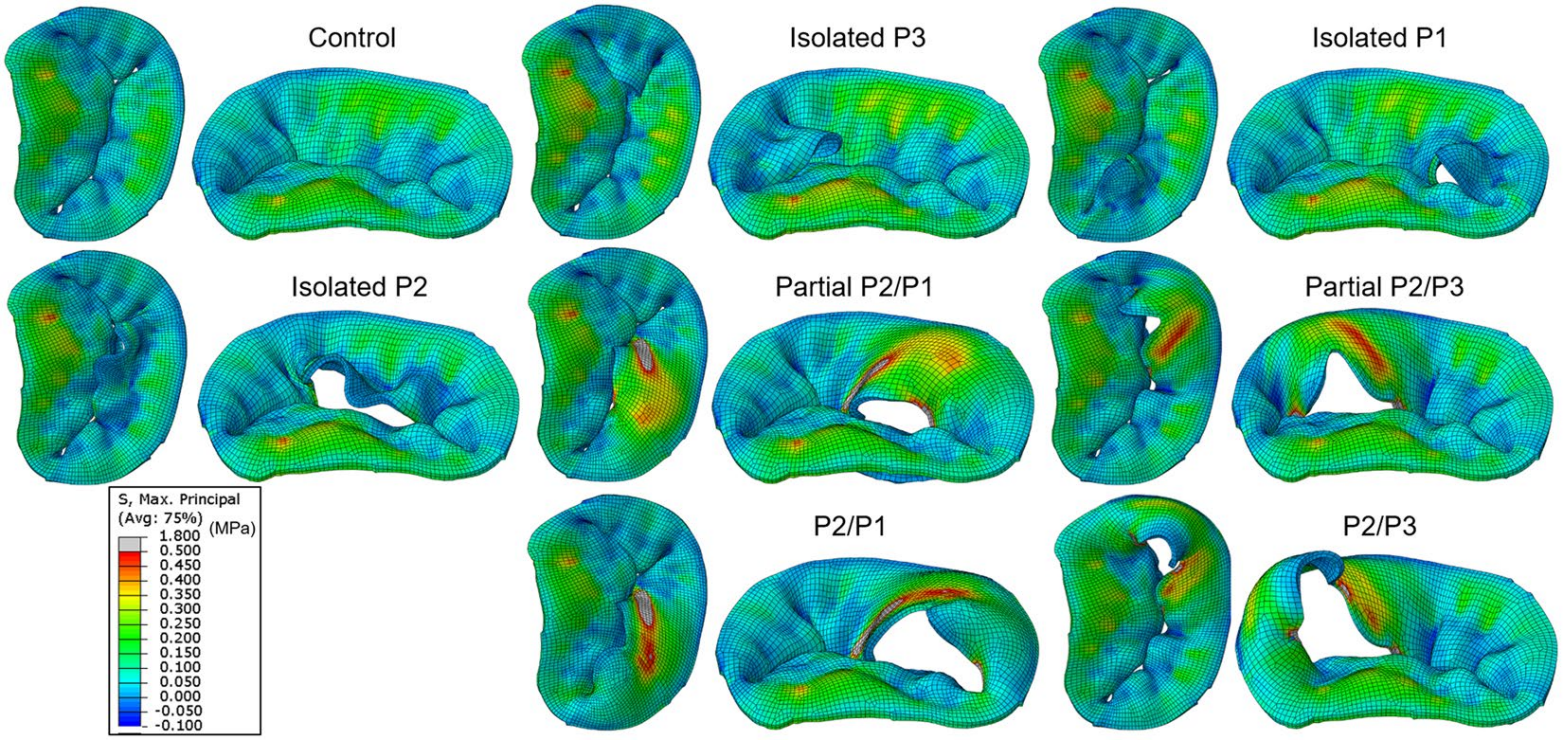
Stress distribution in the mitral leaflets at peak systole.

Figure 3 shows the maximum principal stress distribution across the mitral leaflets at peak systole. A stress value threshold of 0.5 MPa was applied such that relatively large stress values were displayed in grey, facilitating the comparison between all models.

#### Control

As shown in Fig. 3, full leaflet coaptation was clearly observed in the control model, as this was confirmed by the computed MV leakage volume of 0.62 ml. In the PML, high stress regions were observed in the P2 belly to basal region, with a maximum value of 0.34 MPa at the insertion of the basal chordae. Lower levels of tensile stress were found in the P1 and P3 scallops, even resulting in compressive stresses from the bucking or folding of the tissue. In all MV models, a similar pattern of stress distribution was observed in the AML, displaying large stresses in the belly region and close to the fibrous trigones along the MA, regardless of the presence of leaflet prolapse. For the control model, the maximum AML stress was 0.43 MPa, and was located close to the right fibrous trigone region and at the insertion of the strut chordae.

#### Chordae rupture models

PML stress distribution for the chordae rupture models differed from the control model. In the isolated scallop prolapse models, the free edge of the scallop was unrestricted and prolapsed towards the LA, whereas the belly region bent. However, an increase in the stress of the prolapsing scallop was not evident, and even seemed to become less tensile and more compressive. Between the three isolated scallop models, the maximum PML stress value (0.46 MPa) occurred in the isolated P2 model, and was localized near the P2/P1 junction region where the remaining intact basal chordae were connected.

**Figure 4:**
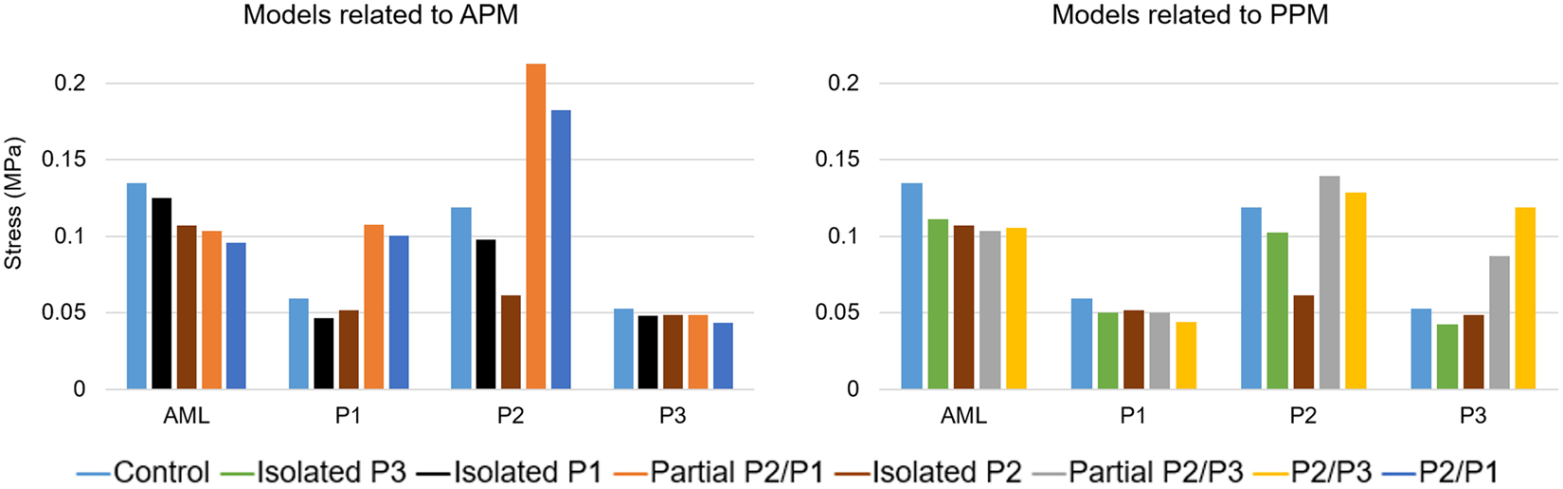
Averaged max. principal stress (MPa) in the mitral leaflets at peak systole.

In the presence of partial or total double scallop prolapse, abnormal bulging of the PML was clearly found in all models. Overall, a broad band of large stresses (0.5–1.8 MPa) occurred in the belly region of the prolapsed segment. Additionally, high stress values (1.5–2.8 MPa) occurred near the scallop junction free edge regions, where the adjacent remaining marginal chordae were connected. Between the four double scallop prolapse models, the maximum stress (2.8 MPa) occurred in the P2/P3 model, and was localized near the free edge of the P2 scallop where the remaining intact marginal chordae were attached. In order to quantitatively compare the stress in the leaflets, Fig. 4 shows the maximum principal stress values averaged over the four sub-regions of the mitral leaflets. Leaflet coaptation and mitral annular regions were not included for the averaged stress calculation.

### LH hemodynamics

**Figure 5:**
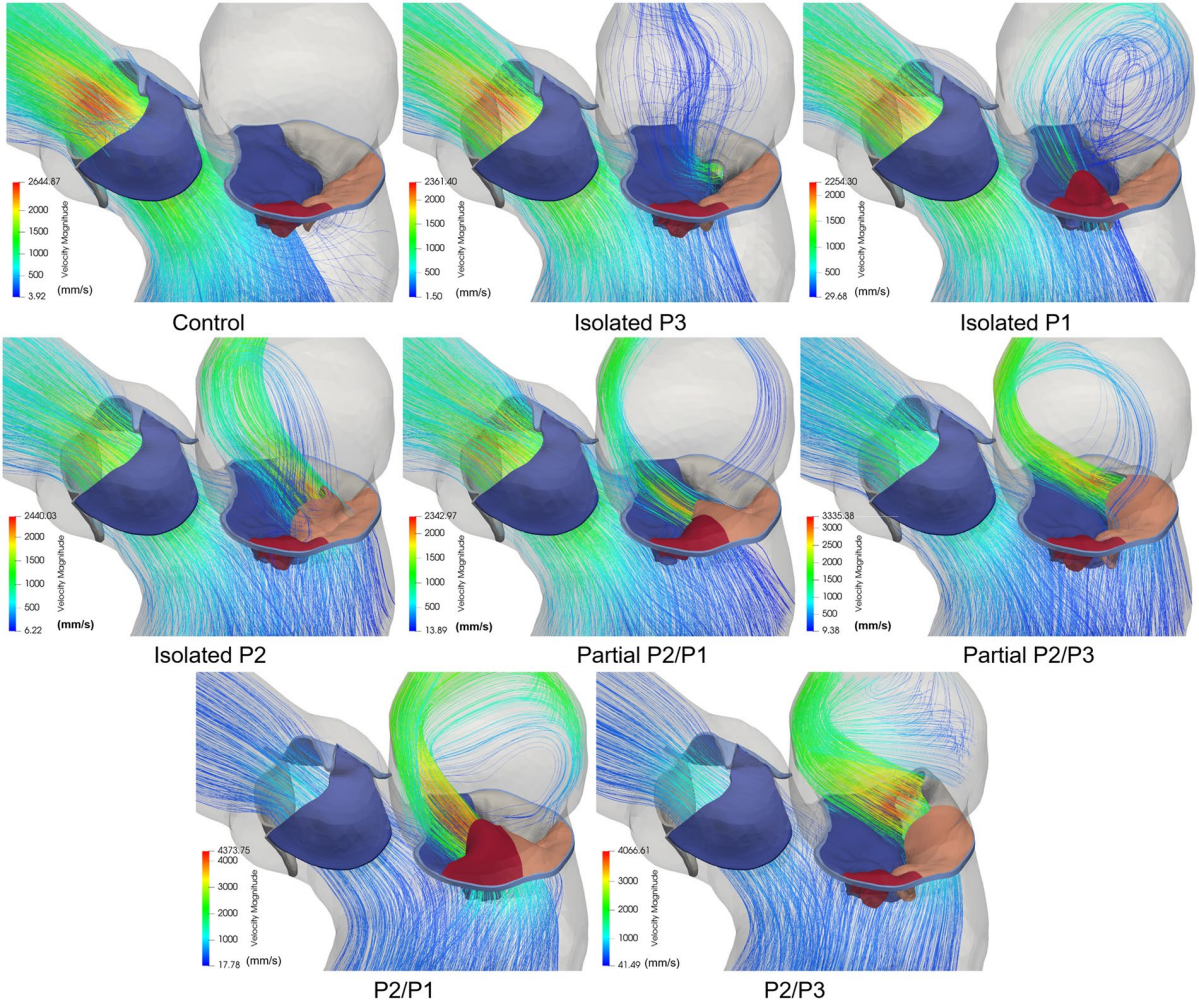
Velocity streamlines showing regurgitant jet structures at peak systole.

Figure 5 shows the 3D velocity streamlines colored by velocity magnitude at peak systole. From the figure it is evident that chordae rupture causes a regurgitant jet into the LA, however, the structure and strength of this jet varies depending on the location and severity of the prolapse. Based on the jet direction, three types of MR jets were found: 1) an anterolaterally directed regurgitant jet was originated in the isolated P3 and P2/P3 models; 2) an anteromedially directed MR jet was found in the isolated P1, isolated P2, and P2/P1 models; and 3) an anteriorly directed central jet was developed in the partial P2/P1 and partial P2/P3 models. These 3D morphologies of the MV and flow structures obtained from the FSI simulations corresponded well to pathologic MR characteristics observed in 2D/3D echo and cardiac magnetic resonance imaging (MRI) data^8,9,11,21^. Additionally, Fig. 5 shows that chordae rupture can produce eccentric and non-eccentric MR jets. An eccentric “wall-hugging” jet that impinged the LA wall was visible in the 4 double scallop prolapse models. Conversely, a non-eccentric jet with more visible 3D flow features and flowing towards the central portion of the LA was found in the 3 isolated scallop prolapse models. Peak regurgitant velocity as well as peak aortic velocity for all models are presented in Table 2. Note that peak regurgitant velocity may not necessarily occur at peak systole.

**Table 2:** Summary of aortic and regurgitant jet velocities and LV efficiency.

	Control	Isolated P3	Isolated P1	Partial P2/P1	Isolated P2	Partial P2/P3	P2/P3	P2/P1
Peak aortic velocity (m/s)	2.69	2.41	2.31	2.38	2.23	2.07	1.33	1.19
Peak regurgitant velocity (m/s)	N/A	5.05	5.01	5.47	4.71	5.71	5.17	5.47
*FSW* (J)	0.89	0.84	0.78	0.74	0.63	0.60	0.31	0.24
*SW* (J)	1.02	1.01	1.01	1.00	0.98	0.98	0.93	0.91
LV efficiency (%)	75.64	71.82	67.59	63.98	55.93	53.20	28.64	22.86

### LV efficiency

**Figure 6:**
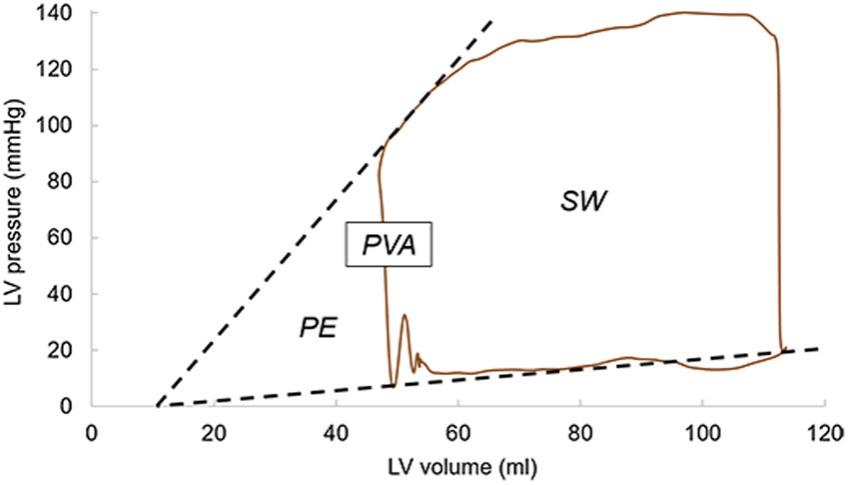
Representative pressure-volume loop for the isolated P2 model.

LV efficiency, an important measure of ventricular pump performance, is defined as the efficiency of energy transfer from the LV to the arterial system^22^. This variable represents the percentage of total energy expenditure by the myocardium that is converted into external stroke work (*SW*). LV efficiency was calculated as *fSW*/*PLA*, where *fSW* is the forward *SW*, and was defined as the time integral of the continuous product of flow across the AV and the aortic pressure during systole^23^. Figure 6 shows a representative pressure-volume loop for the isolated P2 model. As seen in this figure, *PVA*, known as the pressure volume area, represents the total mechanical energy generated by ventricular contraction, where *PVA* = *PE* + *SW*^24^. *SW* corresponds to the area of the pressure-volume loop, and characterizes the external mechanical work achieved by the heart to eject the *LVSV*. *PE*, or potential energy, on the other hand, is the mechanical energy that is available in the LV at end-systole that was not converted into external work and will be dissipated during relaxation^24^. LV efficiency results are summarized in Table 2. As expected, the highest LV efficiency was found for the control model, with a value of 75.64%. MR due to ruptured chordae caused a reduction in the LV pump performance, from 71.82% in mild MR, down to 22.86% in severe MR.

## Discussion

### Mitral apparatus force balance

To the authors' knowledge, this study is the first to quantify and analyze the mitral apparatus force redistribution following chordae rupture using an integrated LV-MV-AV FSI model during the entire cardiac cycle. The results of this study showed that basal/strut chordae carried the majority of the systolic load acting on the leaflets (Fig. 2). Further, basal/strut load decreased as the number of ruptured chordae increased. This reduction in the load was more than 50% for basal chordae when partial or total double scallop prolapse occurred, and more than 20% for strut chordae during total double scallop prolapse. This marked reduction in basal/strut chordae load, together with the corresponding increase in the marginal/intermediate chordae load could be caused by the reduction of the leaflet coaptation zone. A smaller coaptation area increases the tension of the remaining marginal/intermediate chordae, as they have to account for a higher pressure-bearing systolic load that otherwise would be absorbed by proper leaflet apposition. As it has been shown that the thinner marginal chordae are less extensible^25,26^ and weaker than basal chordae^27^, long-term results may lead to chordae elongation, further exacerbate chordae rupture, and eventually cause valve failure.

Chordae tension has previously been measured *in vivo* and *in vitro* under physiological and pathological conditions^28^. However, due to inherent procedural difficulty and in order to avoid interference with normal valve dynamics, these studies have been unable to simultaneously measure the forces across all chordae groups, and instead, have averaged the tension measured in few chordae. Overall, these studies have found that the average tension in a single AML strut chord is the highest among all chordae groups, and that individual marginal chorda force is lower than strut/intermediate chordae forces^29,30^. When the chordae tensions for each chordae group were averaged in our control model, our simulations results seem to agree with the experimental findings, showing that a single AML strut chord is subjected to the highest average load, with a value of 0.5 N. Furthermore, PML and AML marginal chordae carried the lowest average loads, with values of 0.05 N and 0.12 N, respectively. In MV FE models with isolated P2 prolapse developed by Sturla *et al*.^11,12^, the total tension in the chordae located in the proximity of the prolapsing region ranged between 2.56 N and 3.99 N. The present FSI study showed, however, that marginal and intermediate chordae tensions in the isolated P2 prolapse model were 0.52 N and 0.43 N, respectively (see Supplementary Table 1). The discrepancy between our results and other computational studies could be due to differences in the numerical approaches, geometry, material properties, and boundary conditions.

The measurement of the PM forces is critical for the understanding of the mitral apparatus force balance and redistribution under pathological conditions. As seen in Supplementary Table 1, the tension in the PM from where the chordae ruptured was reduced between 10–17% in the 3 isolated scallop prolapse models, more than 38% during partial double scallop prolapse, and around 60% during total double scallop prolapse. Contrary to our results, Sturla and colleagues^11^ noticed a reduction in the PM force from a prolapsed MV to a repaired MV with neo-chordae, which should resemble a healthy MV. At peak systole, it is expected that the PM forces in a physiologic MV should be higher than of a prolapsed MV due to the larger leaflet area experiencing the transmitral pressure difference. Further, Sturla *et al*.^11^ found that the combined PM tension under isolated P2 prolapse ranged between 13.58 N and 17.57 N, which is 30–70% larger than the value found in our isolated P2 prolapse FSI model (10.17 N). In order to have a better understanding of the distribution of the force carried by the PM among the different chordae groups, Table 3 shows the percentage of the force carried by the chordae relative to the total PM force. For the control model, it can be seen that for both PM, the PML chordae carried between 54–60% of the load. This distribution was not strongly affected in the isolated scallop prolapse models. Moreover, when partial or total double scallop prolapse occurred, the opposite PM did not show a significant increase or reduction in its tension, as the total load carried by the PML and AML chordae remained approximately the same as in the control model.

**Table 3:** Summary of distribution of the force (%) carried by the PM among the different chordae groups at peak systole.

	(%)	Control	Isolated P3	Isolated P1	Isolated P2	Partial P2/P1	Partial P2/P3	P2/P3	P2/P1
APM	AML marginal	16.11	16.53	17.04	16.18	15.07	15.00	16.24	49.44
	AML strut	29.94	29.27	30.41	29.73	42.94	26.36	25.74	49.78
	PML marginal	5.26	4.80	0.90	5.26	16.49	16.99	17.94	0.10
	PML intermediate	12.00	12.02	6.35	7.92	15.52	10.64	10.21	0.31
	PML basal	36.70	37.38	45.29	40.90	9.98	31.02	29.87	0.37
PPM	AML marginal	10.88	14.65	18.42	17.63	13.06	21.84	45.47	14.27
	AML strut	28.86	30.21	30.20	32.37	24.92	46.90	53.96	24.46
	PML marginal	6.55	4.55	5.56	4.91	4.34	13.97	0.27	5.00
	PML intermediate	6.43	5.73	5.56	0.14	23.50	0.04	0.08	24.76
	PML basal	47.28	44.86	40.27	44.96	34.18	17.26	0.22	31.50

This capability to systematically quantify the force redistribution from the mitral leaflets to the PM from a healthy to a pathological state is a valuable tool that could lead to an improved understanding of how different pathologies and repair techniques influence LH dynamics and valve mechanics. For example, the use of neo-chordae has made a great number of complex MV pathologies amenable to repair rather than replacement^31^. Nevertheless, determining the optimal number, location and proper length of neo-chordae remains a challenge^12^. As shown by an elegant FE study by Sturla and colleagues^11,12^, small differences in the neo-chordae technique used can significantly alter native chordae forces and leaflet stresses, despite comparable macroscopic successful clinical outcomes. Neo-chordae placement should aim to bring the coaptation region back into the LV inflow region, prevent post-repair systolic AML motion, and overcome MR. Thus, FSI models are required in order to quantify the flow across the valve and accurately simulate full dynamic valve behavior^13^, both of which are essential in the simulation of MV repair because valvular dynamics are affected by the reduction of the effective valvular orifice and restricted motion of the leaflets themselves. The FSI modeling framework developed in this study could overcome some of the weaknesses of the decoupled FE approach, and open the way to assess the coupled LV-valve fluid and structural mechanics before and after MV repair. Combined with clinical data, FSI computational models can provide highly controlled and quantitative analyses of the distinct effects of various valve repair/replacement techniques, pathophysiologic conditions, and anatomical variations on the coupled LH dynamics, and are thus advantageous over FE- or fluid-only computational models, and complementary to *in vivo* and *in vitro* models.

The development of these detailed cardiac computational models for functional evaluation and treatment planning is highly dependent on available clinical imaging modalities, since capturing anatomically accurate 3D images of the cardiac structures is a critical first step of any computational modeling endeavor. Currently, echo is the standard noninvasive clinical imaging modality to examine the cardiac function and valvular diseases. However, due to its limited spatial resolution, patient-specific detailed mitral chordae structure and distribution cannot be accurately captured or segmented^32^. Other noninvasive 3D cardiac imaging modalities include cardiac MRI and multi-slice computed tomography (MSCT). As shown in this study, high-quality MSCT images can enable accurate and detailed segmentation of the valvular and sub-valvular structures of the LH. In case of low MSCT image quality, a complementary segmentation approach that uses optimization methods, parametric modeling using anatomic landmarks points of the valvular apparatus, or generalized anatomical models from literature-derived data, as commonly used in echo-derived computational models, would be required^33^. Fortunately, recent developments in MSCT technology now allow rapid acquisition of volumetric datasets with high spatial and temporal resolution and unlimited 2D planar reconstruction post-processing capability. These new-generation MSCT scanners, together with a parallel rapid expansion of percutaneous valvular repair and replacement techniques have not only allowed that MSCT becomes the standard imaging modality in pre-procedural assessment, but that this imaging modality has the potential to play an important role in the diagnosis and evaluation of valvular pathologies^34^.

### Leaflet stress distribution

In the chordae rupture models where partial or total double scallop prolapse occurred, overall, a high-stress concentration was found in the prolapsed PML segment when compared to the control model. Our simulation results seem to agree with previous computational studies that have compared MV stresses under chordae rupture and normal/repaired states^9,35,36^. For example, Kunzelman *et al*.^6^ investigated MV dynamics under physiological and P2 chordae rupture states. Similar to our study, it was found that the leaflet stress increased during chordae rupture, and that stress concentrations were located close to the attachment point of the adjacent remaining native chordae. Similarly, Sturla *et al*.^11^ found that the stress in the prolapsed P2 scallop increased during chordae rupture, indicating excessive loading of the remaining chordae and adjacent leaflet tissue. Nevertheless, as seen in Fig. 4, our study showed that chordae rupture in a specific scallop does not always involve an increase in the stress of the entire leaflet. For example, in the P1 and P3 isolated models, it was found that the average stress in the prolapsed scallop in fact decreased around 20%, while in the neighboring and opposite scallops decreased between 9–18%. Moreover, in the isolated P2 model, the reduction in the stress of the P2 scallop was 50%, while in the neighboring scallops (i.e. P1 and P3) decreased between 8–13%.

On the contrary, in the four models where partial or total double scallop prolapse occurred, the stress in the lateral (P1 or P3) scallops increased more than 65%, in the central (P2) scallop increased between 8–80%, and in the healthy scallop decreased between 7–25%. It is noteworthy that the average stress in the AML was also reduced as the number of ruptured chordae increased. This reduction was more than 20% when partial or total double scallop prolapse occurred. This indicates that PML chordae rupture also affects the stress distribution in the AML. It is reasonable to expect that these differences in tissue loading can cause the mitral leaflets to remodel and adapt to the geometric needs imposed by chordae elongation and rupture^37,38^, similar as chronic tension leads to permanent increases in size in bone, vessels, and skin^39–41^. However, little is known about this phenomenon, in part due to the lack of a measuring technique that can accurately and parametrically quantify the MV mechanical state under different pathophysiological loads. The FSI modeling framework developed in this study can potentially allow for such measurements, which can provide invaluable data for exploring the basic mechanisms of tissue remodeling.

### Pathological LH hemodynamics

Accurate grading of MR severity is crucial for clinical decision making, prognosis, and timing of treatment intervention. The most common method for noninvasive assessment of MR is echo, however, accurate and reproducible quantification of the *RV*_*MV*_ is compromised by the presence of multiple jets, the dynamic regurgitant orifice, and geometrical assumptions of the mitral orifice and the LV outflow tract (LVOT)^42^. Moreover, given the possible eccentricity of the regurgitant jet into the LA with a marked Coanda effect, quantification of MR severity can be underestimated due to limitations of alignment, hydrodynamic principle assumptions using the PISA method, and Color Doppler jet size attenuation when the jet is wall hugging^43^. Herein the importance of quantifying the *RV*_*MV*_ with a volumetric technique, that although is time consuming as it requires calculation of the *SV* and *RV* through the regurgitant and reference valves, has advantages in eccentric MR jets, multiple jets, and mid-to-late systolic MR.

As shown in Tables 1 and 2, the modeling approach implemented in this study was able to assess MR severity by directly quantifying the *RV*_*MV*_, which is a strong indicator of MV function, as well as its effect on the aortic blood flow. Moreover, this is the first computational work that reported different MR jet structures in the LA using an FSI framework. As seen in Fig. 5, while some regurgitant jets followed the direction of the flow determined by the prolapsed segment; with a more central location, other Coanda jets appeared to be attracted towards the atrial wall. The results of this work could be used to better understand how the regurgitant flow features can affect LA enlargement^44^, as well as echo assessment of MR by performing a systematic comparison of the different semi-quantitative and quantitative techniques. Heart pumping efficiency is also an important hemodynamic metric that can be studied with an integrative computational approach. When MR was present, there was a progressive reduction in the LV pump efficiency in performing *fSW*, since the total SW was divided into the *fSW* and the ineffective work of the volume regurgitation into the LA. Impaired LV efficiency for the chordae rupture models was between 71.82-22.86%. Our model predictions seem to agree well with *in vivo* measurements under healthy and MR conditions^45^. For example, Kameyama *et al*.^24^ found that the human LV efficiency is 69% ± 10% under physiological conditions.

It is also important to note that besides the expected increase in the MV leakage volume as the number of ruptured chordae increased (see Table 1), a higher MV closing volume was also predicted; especially in the severe MR models. It was found that although the degree of MV opening and the timing of valve closure was similar for all LH models at end-diastole, the mitral leaflets coapt together faster in the control model. The reason behind this is that the basal chordae were the first to carry the pressure load early in systole, preventing the base of the PML to partially bulge upward into the LA due to the increased transmitral pressure, as it occurred in the LH models with partial or total double scallop prolapse. In the control model, this allowed that a larger leaflet area was perpendicular to the pressure gradient, therefore creating a larger closing force and causing the leaflets to coapt together faster. This finding underscores: a) the importance of basal chordae in optimizing MV closure, and b) the fact that blood flow dynamics and the structural mechanics of the valves should be modeled together using FSI in order to capture the full valve dynamics throughout the cardiac cycle.

In this study, we make a first attempt in relating the ventricular fluid dynamics with the valve mechanics during MR by modeling flow-leaflet interaction, leaflet coaptation, and flow dynamics into, outward and within the LV. Studies like this are significant because it is known that for complex systems such as the heart, the structural deformation of the valves is intricately associated with the blood flow dynamics, and a deeper understanding of the interaction between the different components may lead to useful tools to diagnose and treat any abnormality of one part based on available information about the other. Performing FSI simulations of the LH dynamics to a satisfactory level, however, is not a trivial task. The main challenges to overcome are: 1) the large nonlinear deformation of the valve leaflets, 2) flow domain discontinuity due to rapid valve closure, and 3) the computation of flow-induced loads on evolving complex fluid-solid interfaces. Regardless of significant advances in the field, existing FSI methods using either a boundary conforming formulation such as the Arbitrary Lagrangian Eulerian (ALE), or a non-boundary conforming formulation such as the immersed boundary method (IBM) experience several complications^46,47^, mainly because the fluid mechanics are most conveniently described using the Eulerian formulation, while the solid equations are normally described in the Lagrangian formulation. Using a Lagrangian description for both fluid and solid domains is a potential remedy to the simulation of FSI problems, and smoothed particle hydrodynamics (SPH) is one of these methods. The meshless nature of SPH allows large deformations to be modeled more easily in cases where mesh-based methods would require dealing with expensive re-meshing and with a moving interface on a stationary mesh. Previous studies have concluded that the SPH method is a viable FSI technique to simulate the complex valve dynamics and the large-scale ventricular flow dynamics^14,17,18,48,49^. Nevertheless, like any other numerical method, SPH has its own limitations and further developments are still required for SPH to compete with established mesh-based methods. For example, in this study each cardiac cycle required approximately 5 days to run. As a result, the present FSI modeling framework cannot be used in a clinical setting and currently, is only suitable for a research environment. However, both FE and SPH solvers can use the GPU’s extremely parallel architecture^15,50,51^, which will significantly reduce the running time in the near future and avoid the need for a large computer cluster.

### Limitations

There are some limitations that should be taken into account when interpreting the results of this study. First, in this parametric FSI study we adapted one previously validated healthy LH model to create seven chordae rupture cases, employing the same baseline conditions for all diseased models, with variations of chordae rupture location and number. Such well-controlled, side-by-side comparisons are difficult to obtain in real patient conditions. However, we acknowledge that a large cohort of patient-specific LH models needs to be developed and simulated to replicate clinical conditions and draw statistical conclusions. Second, there is no direct validation of the chordae rupture LH models. We assume that LH and valve computational models that have been validated under physiological conditions^17,18,52^ can also accurately simulate some pathological states, such as acute MR due to chordae rupture. To allow for validation of the chordae rupture models, a study of patients with mitral leaflet prolapse scheduled for MV repair would be needed, and clinical measurements should be compared to FSI results.

Third, the FSI modeling protocol adopted in this study required a considerable amount of human effort to create the LH models and of computational time to run the FSI simulations, which is not compatible with its use in a routine clinical setting. We are currently developing methods to streamline and automate the model generation and simulation process to facilitate analysis of larger patient cohorts^53^. Fourth, although alterations in tissue properties have been found when MV prolapse is caused by chordae rupture^54^, the same mitral tissue properties and geometry (e.g. tissue thickness) were used for all models in this study. Therefore, simulation results may differ if patient-specific pathological tissue and geometrical data are incorporated. Finally, due to the inherent limitation of Abaqus SPH formulation, small-scale flow and turbulence features may not be accurately solved close to the cardiac wall. When particles are close to the wall, part of the supporting domain of the smoothing kernel will not be filled with SPH particles, affecting their integration accuracy. However, the combined effect of the smoothing kernel interpolation function near the wall and the node-to-surface contact interaction partially enforces the no-slip boundary condition.

## Conclusions

This study investigated the impact of PML chordae rupture on LH dynamics and severity of MR. By using a novel FSI numerical framework together with an integrated LH model that considered both the MV and AV, we performed a parametric computational study that simultaneously quantified and analyzed chordae forces, PM forces, leaflet stresses, intraventricular hemodynamics and cardiac efficiency in the sequential transition from a physiological state to severe acute MR through multiple levels of chordae rupture. A detailed mechanistic understanding of the mitral apparatus force redistribution and coupled fluid and structural LH dynamics can give deeper insight into the pathophysiologic developmental mechanisms of MV prolapse due to chordae rupture, allow for evaluation and prediction of interventional treatments, and ultimately support improved clinical outcomes.

## Methods

**Figure 7:**
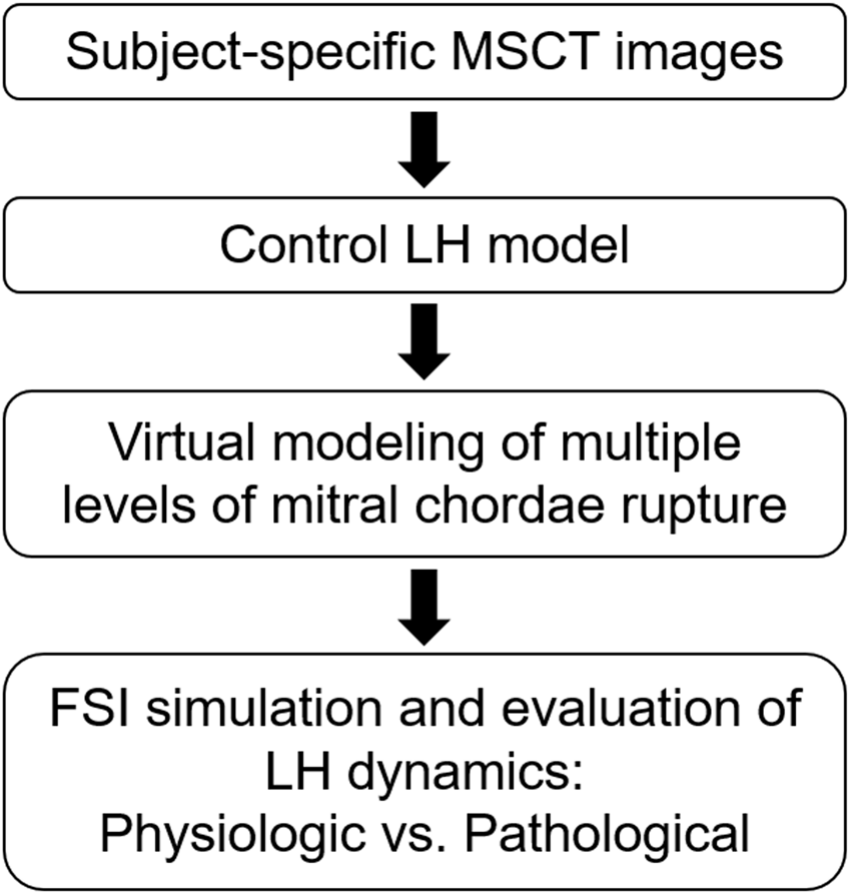
Computational modeling protocol for investigation of LH dynamics following mitral chordae rupture.

Figure 7 presents our computational modeling protocol for investigation of LH dynamics following mitral chordae rupture. This protocol was composed of FSI simulation of physiologic LH function (control), virtual modeling of multiple levels of chordae rupture, FSI simulation of LH function after chordae rupture, and evaluation of the functional and biomechanics characteristics of the coupled LV-valve pathological dynamics.

### LH model creation

**Figure 8:**
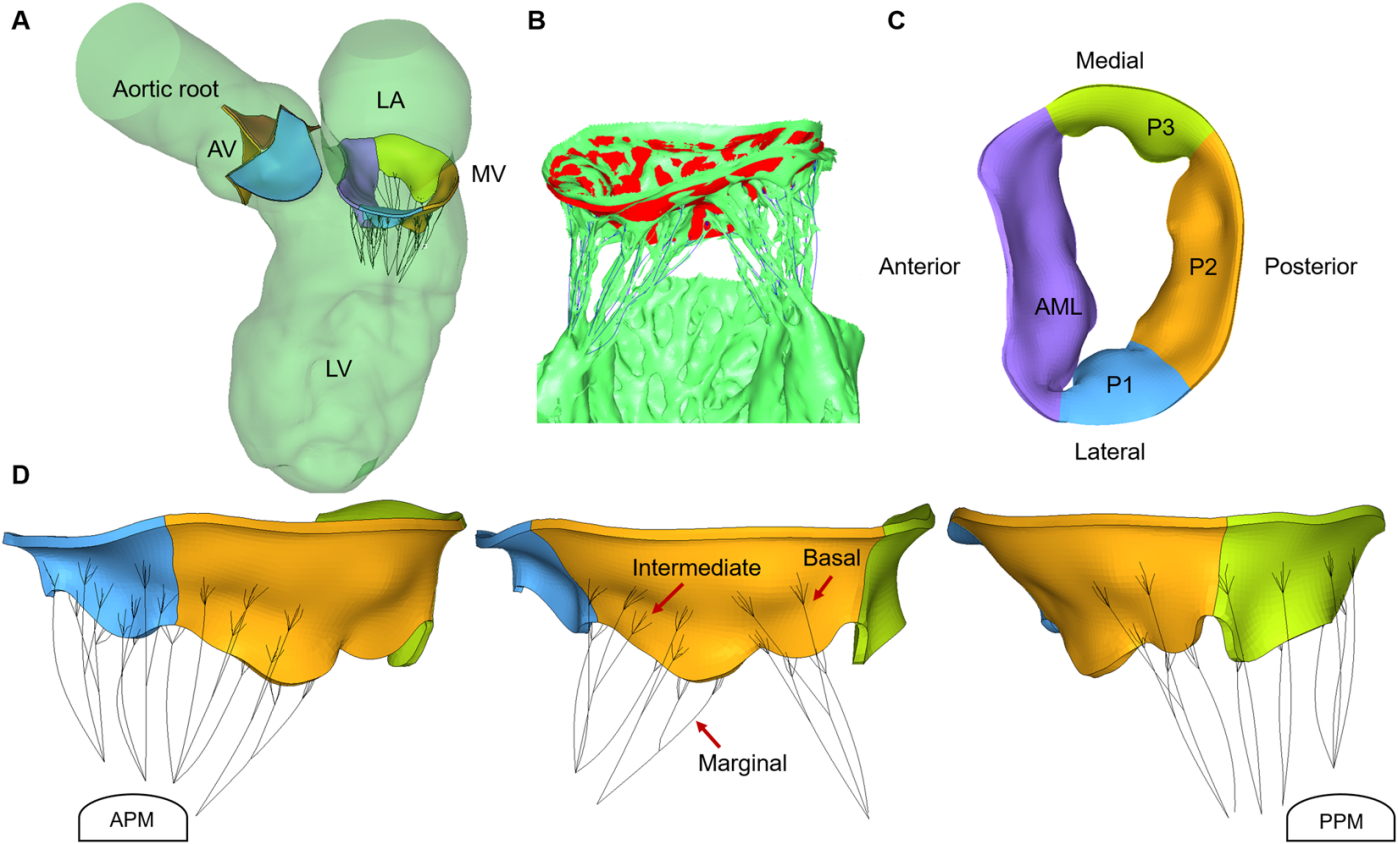
(**A**) LH model, (**B**) MV model reconstructed from MSCT images, (**C**) top view of MV with four sub-regions, (**D**) PML chordae groups. AML chordae not shown for clarity.

In this study, we employed a previously developed LH FSI model with normal cardiac function and no heart valve abnormalities. This control LH model, validated against subject-specific transthoracic echocardiography data, was shown to capture the physiological bulk intraventricular hemodynamics, valves nonlinear elastic response and leaflet kinematics during the full cardiac cycle^17,18^. Briefly, cardiac MSCT images of a 72-year-old female patient referred for transcatheter AV replacement were collected from Hartford Hospital (Hartford, CT). The use of de-identified patient clinical data for this study was approved by an Institutional Review Board. The MSCT examination was performed on a GE LightSpeed 64-channel volume CT scanner with an in-plane resolution of 0.82 × 0.82 mm and a slice thickness of 0.625 mm. Image segmentation was performed using Amira-Avizo (Thermo Fisher Scientific, MA) and 3D Slicer (www.slicer.org) software, while HyperMesh (Altair Engineering, Inc., MI) software was used for mesh generation. As seen in Fig. 8A, the reconstructed LH model comprises all major cardiac structures, including the aortic root, AV, LV, MV and proximal left atrium (LA). The AV calcification observed in the MSCT images was not incorporated in the LH model in order to mimic a healthy state. End-diastolic (EDV), end-systolic (ESV), stroke volume (SV) and ejection fraction (EF) values were 113 ml, 47 ml, 66 ml and 58%, respectively.

A semi-automated segmentation method previously developed was used to extract the main cardiac structures of interest^17,18^. Briefly, cardiac wall and valvular geometries were identified and manually segmented from the rest of the heart structures using Hounsfield intensity thresholding and region growing techniques for the cardiac wall, combined with the brush tool and manual pixel editing for the valve leaflets and subvalvular components, making sure to digitally dissect and clearly differentiate individual mitral chordae. Next, smoothing and wrapping operations such as islands editing, removing extrusions, filling small holes and specific automatic smoothing modules were used to fix inconsistencies in the model due to image noise and improve the mesh quality. Altogether, approximately 30 hours of work were required to segment the MSCT data, including segmentation of the 10 cardiac frames for the cardiac wall. The AV and MV geometries were segmented at mid-systole and mid-diastole, respectively, approximating the stress–free configuration^52,55^. A constant thickness of 0.5 mm was given to the AV leaflets, while the MV leaflets had locally varying thickness with average values of 1 mm and 1.2 mm for the leaflet belly and edge regions, respectively. The MV model used in this study was developed in a previous work from our group that validated healthy MV dynamics by quantitatively comparing the closed valve shape from FE simulation with the MSCT images during systole^52^.

As seen in Fig. 8B, the detailed mitral chordae structure (number, position, length, branching, origins of the PM tips, and insertions into the leaflets) was clearly visible from the MSCT images, which had excellent clarity and crisp detail^52^. Chordae were classified into five groups according to the respective insertion zone on the leaflets: anterior strut, anterior marginal, posterior marginal, posterior intermediate, and posterior basal^28^. As shown in Fig. 8D, marginal chordae insert into the leaflet free edge, intermediate chordae insert on the leaflet rough zone, and basal chordae insert closer to the leaflet base. High-quality images also allowed us to identify and delineate the chordae that protruded from the PM heads. Simple point insertions were then used to model the distribution of the chordae origins on the PM tips. Identifying and creating the chordae to leaflet transition zone, on the other hand, is a nontrivial problem, since chordae seamlessly fuse into the leaflets and limited image resolution can prevent the complete detection of this detailed structure. For the basal and strut chordae, the leaflet-chordae transition zone was easily identified from the MSCT images. Fork-shaped truss elements were used to follow the visible chordae splits and avoid local stress concentrations on the leaflets. For some of the intermediate and marginal chordae, however, the transition region was not clearly detected. Thus, the number of splits and their lengths were initially estimated and required further manual refinement. This optimization process aimed to quantitatively match the FE deformed MV systolic geometry with the subject-specific MSCT images^52^. In summary, chordae originated from the anterolateral PM (APM) and posteromedial PM (PPM) tips, and distributed to the ventricular surface of the leaflets as presented in Table 4. A total of 17 chordae origins were modeled from the PM tips. Cross-sectional area values of 0.71 mm^2^, 2.05 mm^2^, and 0.38 mm^2^ were assigned to basal/intermediate, strut, and marginal chordae, respectively^52^. Each chord was modeled with up to 10 elements, with an average element length of 1.5 mm.

**Table 4:** Mitral chordae structure.

		From APM	From PPM
Number of chordae origins	AML	5	3
	PML	4	5
Number of chordae leaflet insertions	AML marginal	8	6
	AML strut	3	4
	PML marginal	7	6
	PML intermediate	7	1
	PML basal	5	6

### Simulated MV conditions

The control LH model was defined as having all mitral apparatus anatomical structures intact. PML prolapse is more prevalent than AML prolapse^56^, usually involving ruptured chordae in the central scallop (P2), which then extends to the lateral P1 and P3 scallops^11,12,57,58^ (see Fig. 8C). Thus, the chordae rupture LH models were virtually created by modifying the control model, initially removing marginal and intermediate chordae elements in the individual PML scallops, and then progressing towards rupture of the basal chordae; causing partial or total double scallop prolapse. As seen in Table 1, a total of seven chordae rupture LH models were created. In the present study, we strictly followed our previously reported computational modeling protocols for investigation of MV function under healthy and diseased states^52,55,59^.

### Numerical modeling

An FSI modeling approach based on the combination of SPH and nonlinear FE formulation has been adopted in this study^17^. SPH is a fully Lagrangian modeling scheme that allows the discretization of a prescribed set of continuum equations by interpolating the properties directly at a discrete set of points distributed over the solution domain without the need of a spatial mesh^60^. SPH uses an evolving interpolating (kernel) function and its derivatives to approximate a field variable at any point in the domain. The SPH-FE framework was implemented in the commercial software Abaqus/Explicit (SIMULIA, Providence, RI). The coupling between the SPH particles and the structural model is accomplished through normal contact definitions by finding the best penalty force to prevent interface penetration and satisfy conservation of momentum. A density of $\rho =1056\,{\rm{kg}}/{m}^{3}$ and a dynamic viscosity of $\mu =0.0035\,{\rm{Pa}}\cdot {\rm{s}}$ was set for blood properties. Based on a previous particle-sensitivity analysis^18^, the optimum number of particles in the SPH domain was determined to be approximately 498,000 one-node (PC3D) elements. The FE domain was meshed with eight-node hexahedral (C3D8R) solid elements to capture the thickness of the AV and MV, four-node quadrilateral (S4R) elements to represent the cardiac wall, and 3D stress/displacement truss elements (two-node linear T3D2) to model the chordae tendineae. Further details on the FSI framework setup are described in a previous study^17^.

### Material models description

Human leaflet tissue was assumed to be an incompressible, anisotropic, nonlinear, hyperelastic material. Thus, the strain energy function, *W*, given by Eq. 1, can be expressed by a fiber reinforced hyperelastic material model (i.e. MHGO model) based on the work of Holzapfel, Gasser and Ogden^61^

1 $$W={C}_{10}\{\exp [{C}_{01}({\bar{I}}_{1}-3)]-1\}+\frac{{k}_{1}}{2{k}_{2}}\sum _{i=1}^{2}\,[\exp {k}_{2}{[\kappa {\bar{I}}_{1}+(1-3\kappa ){\bar{I}}_{{4}_{i}}-1]}^{2}-1]+\frac{1}{D}{(J-1)}^{2},\,i=1,2$$

where *C*_10_, *C*_01_, *k*_1_, *k*_2_, *k* and *D* are material constants, and ${\bar{I}}_{1}\,$and ${\bar{I}}_{4i}\,\,$are the deviatoric strain invariants. *C*_10_ and *C*_01_ describe the matrix material, *D* is a material constant to impose incompressibility, and *J* is the determinant of the deformation gradient. *k*_1_ is a positive constant with the dimension of stress to describe the fiber material and *k*_2_ is a dimensionless parameter. In addition, *k* describes the distribution of fiber orientation. Local coordinate systems were defined for each leaflet to include local fiber orientation. The anisotropic MHGO material model was implemented into Abaqus/Explicit with a user sub-routine VMAT. Additionally, the isotropic hyperelastic Ogden material model^62^ was used to characterize the mechanical properties of the chordae. The material parameters, given in Supplementary Table 2, were determined by fitting in-house multiprotocol biaxial and uniaxial testing data of healthy human cardiac tissues (80-year-old female). Further details on the determination of material parameters have been described in our previous publications^63,64^.

### Boundary conditions and FSI model solution

**Figure 9:**
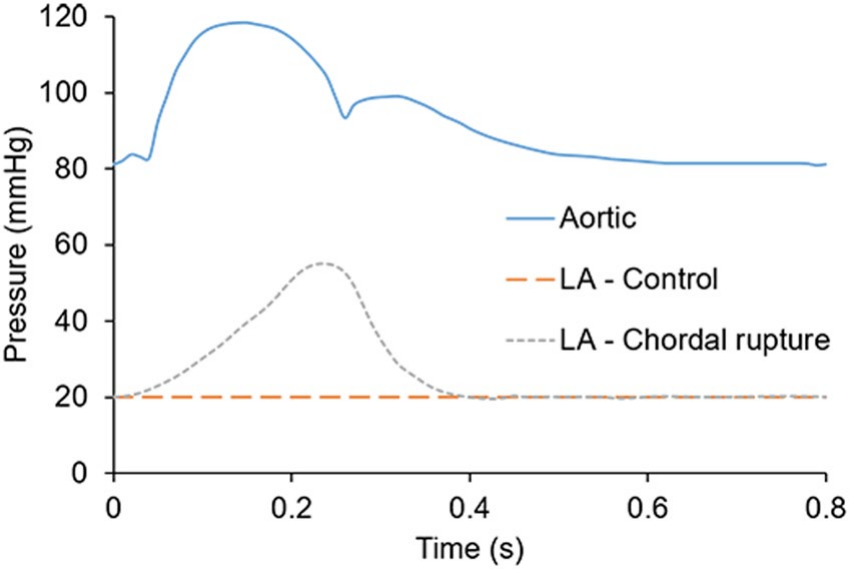
Aortic and LA pressure boundary conditions.

Pressure boundary conditions were applied at the atrial inlet and aortic outlet of the LH models. For the control model, a constant LA pressure of 20 mmHg was prescribed at the inlet, while an aortic pressure waveform, as seen in Fig. 9, was applied at the outlet^17^. In acute MR due to ruptured chordae, *RV*_*MV*_ in a normal-sized LA results in a marked increase in the atrial pressure (elevated V-wave)^65^. Thus, the chordae rupture models employed a pathological atrial inlet pressure waveform^66^, while the same aortic pressure waveform as in the control model was applied at the outlet.

Cardiac wall motion was applied to the LH models as a time-dependent nodal displacement boundary condition based on the Mao *et al*. study^17^. Briefly, each frame of the MSCT dataset (10 cardiac frames) was initially manually segmented and triangulated meshes were obtained by means of 3D Slicer software. Second, from the segmented mesh in end-systole, a high-quality template surface mesh was created and divided in subdomains (LV sac, aortic root, MA, proximal LA) using Hypermesh software. Third, the morphing toolkit available in Hypermesh software was used to deform and map the template mesh to the target mesh from the next MSCT frame. This morphing process was performed sequentially for all cardiac frames and manually tuned. In this fashion, the cardiac wall mesh maintained the same number of nodes across all frames, thus ensuring one-to-one node correspondence. Finally, as numerical simulations involve time steps smaller than the time interval between two MSCT frames, nodal displacements were interpolated with a cubic spline. In this study, the cardiac wall motion was kept the same for all LH models. This approach aims to replicate the instantaneous changes in cardiac dynamics during acute MR due to chordae rupture, when the LV has not yet remodeled, but the afterload is decreased as the result of partial systolic emptying into the LA. Thus, the compensatory and cardiac remodeling mechanisms under chronic MR were not considered. Chordae origins nodes were not directly attached to the PM, but were tracked from the MSCT images and displaced between two spatial locations representing PM tips location during mid-diastole and mid-systole^17^. The beginning of the systolic phase was selected as the starting point of the simulation, resembling the isovolumetric contraction phase. The patient’s heart rate was 75 bpm, corresponding to a cardiac cycle of 0.8 s. Two cardiac cycles were conducted and the results from the second cycle were analyzed^17^. SPH particle sensitivity^18,67^ and valve mesh sensitivity^52^ studies were previously performed. Simulations were run on an Intel Xeon E5-2670 cluster with 64 cores and required approximately 240 hours to run two cardiac cycles.

## Electronic supplementary material

Supplementary Information

## Data Availability

All relevant data are within the paper.

## References

[1] Benjamin EJ (2017). Heart disease and stroke statistics—2017 update: a report from the American Heart Association. Circulation.

[2] Gabbay U, Yosefy C (2010). The underlying causes of chordae tendinae rupture: a systematic review. International journal of cardiology.

[3] Grande-Allen KJ, Ratliff NB, Griffin B, Cosgrove D, Vesely I (2001). Case report: outer sheath rupture may precede complete chordal rupture in fibrotic mitral valve disease. The Journal of heart valve disease.

[4] Nelson, J. S. & Bolling, S. F. In *Seminars in thoracic and cardiovascular surgery*. 1–4 (Elsevier). 10.1053/j.semtcvs.2012.01.008 22643652

[5] Sun W, Martin C, Pham T (2014). Computational modeling of cardiac valve function and intervention. Annual review of biomedical engineering.

[6] Kunzelman K, Reimink MS, Verrier ED, Cochran RP (1996). Replacement of mitral valve posterior chordae tendineae with expanded polytetrafluoroethylene suture: a finite element study. Journal of cardiac surgery.

[7] Reimink M, Kunzelman K, Cochran R (1996). The effect of chordal replacement suture length on function and stresses in repaired mitral valves: a finite element study. The Journal of heart valve disease.

[8] Rim Y, Laing ST, McPherson DD, Kim H (2014). Mitral valve repair using ePTFE sutures for ruptured mitral chordae tendineae: a computational simulation study. Annals of biomedical engineering.

[9] Rim Y, Choi A, McPherson DD, Kim H (2015). Personalized computational modeling of mitral valve prolapse: Virtual leaflet resection. PloS one.

[10] Choi A, McPherson DD, Kim H (2017). Neochordoplasty versus leaflet resection for ruptured mitral chordae treatment: Virtual mitral valve repair. Computers in Biology and Medicine.

[11] Sturla F (2014). Is it possible to assess the best mitral valve repair in the individual patient? Preliminary results of a finite element study from magnetic resonance imaging data. The Journal of thoracic and cardiovascular surgery.

[12] Sturla F (2015). Biomechanical drawbacks of different techniques of mitral neochordal implantation: When an apparently optimal repair can fail. The Journal of thoracic and cardiovascular surgery.

[13] Lau K, Diaz V, Scambler P, Burriesci G (2010). Mitral valve dynamics in structural and fluid–structure interaction models. Medical engineering & physics.

[14] Mao W, Li K, Caballero A, Sun W (2016). Fully-coupled Fsi Simulation of Bioprosthetic Heart Valve Using Smoothed Particle Hydrodynamics. Cardiology.

[15] Toma M (2017). Fluid-structure interaction analysis of ruptured mitral chordae tendineae. Annals of biomedical engineering.

[16] Khodaei S, Fatouraee N, Nabaei M (2017). Numerical simulation of mitral valve prolapse considering the effect of left ventricle. Mathematical biosciences.

[17] Mao W, Caballero A, McKay R, Primiano C, Sun W (2017). Fully-coupled fluid-structure interaction simulation of the aortic and mitral valves in a realistic 3D left ventricle model. PloS one.

[18] Caballero, A. *et al*. Modeling Left Ventricular Blood Flow Using Smoothed Particle Hydrodynamics. *Cardiovascular Engineering and Technology*, 1–15 (2017). 10.1007/s13239-017-0324-z PMC5709227 28744784

[19] Zoghbi WA (2003). Recommendations for evaluation of the severity of native valvular regurgitation with two-dimensional and Doppler echocardiography. Journal of the American Society of Echocardiography.

[20] Nishimura RA (2017). 2017 AHA/ACC focused update of the 2014 AHA/ACC guideline for the management of patients with valvular heart disease: a report of the American College of Cardiology/American Heart Association Task Force on Clinical Practice Guidelines. Circulation.

[21] Torigoe T (2012). Clinical characteristics of acute mitral regurgitation due to ruptured chordae tendineae in infancy—experience at a single institution. European journal of pediatrics.

[22] Rodriguez, D. *Left Ventricular Pressure-Volume Analysis: an example of function assessment on a sheep*, Université Paris Sud (2015).

[23] Rimehaug AE (2013). Cardiac power integral: a new method for monitoring cardiovascular performance. Physiological reports.

[24] Kameyama T (1992). Energy conversion efficiency in human left ventricle. Circulation.

[25] Kunzelman K, Cochran K (1990). Mechanical properties of basal and marginal mitral valve chordae tendineae. ASAIO Journal.

[26] Zuo K (2016). Characterization of biomechanical properties of aged human and ovine mitral valve chordae tendineae. Journal of the mechanical behavior of biomedical materials.

[27] Sedransk KL, Grande-Allen KJ, Vesely I (2002). Failure mechanics of mitral valve chordae tendineae. The Journal of heart valve disease.

[28] Rabbah J-PM, Saikrishnan N, Siefert AW, Santhanakrishnan A, Yoganathan AP (2013). Mechanics of healthy and functionally diseased mitral valves: a critical review. Journal of biomechanical engineering.

[29] Jimenez JH, Soerensen DD, He Z, He S, Yoganathan AP (2003). Effects of a saddle shaped annulus on mitral valve function and chordal force distribution: an *in vitro* study. Annals of biomedical engineering.

[30] Jimenez JH, Soerensen DD, He Z, Ritchie J, Yoganathan AP (2005). Effects of papillary muscle position on chordal force distribution: an *in-vitro* study. J Heart Valve Dis.

[31] Wei, L., Jiang, L. & Li, Y. The use of artificial chordae in mitral valve repair. *Journal of cardiac surgery* (2017). 10.1111/jocs.13120 28303614

[32] Gao Hao, Qi Nan, Feng Liuyang, Ma Xingshuang, Danton Mark, Berry Colin, Luo Xiaoyu (2017). Modelling mitral valvular dynamics-current trend and future directions. International Journal for Numerical Methods in Biomedical Engineering.

[33] Chandran KB, Kim H (2015). Computational mitral valve evaluation and potential clinical applications. Annals of biomedical engineering.

[34] Wunderlich NC (2018). Imaging for Mitral Interventions: Methods and Efficacy. JACC: Cardiovascular Imaging.

[35] Reimink MS, Kunzelman KS, Verrier ED, Cochran RP (1995). The Effect of Anterior Chordal Replacement on Mitral Valve Function and Stresses: A Finite Element Study. Asaio Journal.

[36] Choi A, McPherson DD, Kim H (2016). Biomechanical evaluation of the pathophysiologic developmental mechanisms of mitral valve prolapse: effect of valvular morphologic alteration. Medical & biological engineering & computing.

[37] Rausch MK, Tibayan FA, Miller DC, Kuhl E (2012). Evidence of adaptive mitral leaflet growth. journal of the mechanical behavior of biomedical materials.

[38] Stephens EH (2008). The effects of mitral regurgitation alone are sufficient for leaflet remodeling. Circulation.

[39] Gosain Arun K., Song Lian-Sheng, Santoro Timothy, Weihrauch Dorothee, Bosi Brook O., Corrao Marlo A., Chilian William M. (2000). Effects of Transforming Growth Factor-β and Mechanical Strain on Osteoblast Cell Counts: An in Vitro Model for Distraction Osteogenesis. Plastic and Reconstructive Surgery.

[40] De Filippo RE, Atala A (2002). Stretch and growth: the molecular and physiologic influences of tissue expansion. Plastic and reconstructive surgery.

[41] Davis NP, Han H-C, Wayman B, Vito R (2005). Sustained axial loading lengthens arteries in organ culture. Annals of biomedical engineering.

[42] Little SH (2010). Quantifying mitral valve regurgitation: new solutions from the 3rd dimension. Journal of the American Society of Echocardiography.

[43] Ginghina C (2007). The Coandă effect in cardiology. Journal of Cardiovascular Medicine.

[44] Le Tourneau T (2010). Impact of left atrial volume on clinical outcome in organic mitral regurgitation. Journal of the American College of Cardiology.

[45] Imasaka K-I (2013). Early mitral valve surgery for chronic severe mitral regurgitation optimizes left ventricular performance and left ventricular mass regression. The Journal of thoracic and cardiovascular surgery.

[46] Ghosh, R. *et al*. Comparative Fluid-Structure Interaction Analysis of Polymeric Transcatheter and Surgical Aortic Valves’ Hemodynamics and Structural Mechanics. *Journal of biomechanical engineering* (2018). 10.1115/1.4040600 30029207

[47] Bavo AM (2016). Fluid-structure interaction simulation of prosthetic aortic valves: comparison between immersed boundary and arbitrary Lagrangian-Eulerian techniques for the mesh representation. PloS one.

[48] Toma, M. *et al*. Fluid–structure interaction and structural analyses using a comprehensive mitral valve model with 3D chordal structure. *International Journal for Numerical Methods in Biomedical Engineering* (2016). 10.1002/cnm.2815 PMC5183567 27342229

[49] Shahriari S, Kadem L, Rogers B, Hassan I (2012). Smoothed particle hydrodynamics method applied to pulsatile flow inside a rigid two‐dimensional model of left heart cavity. International journal for numerical methods in biomedical engineering.

[50] Shahriari, S. & Kadem, L. In *Numerical Methods and Advanced Simulation in Biomechanics and Biological Processes* 203–219 (Elsevier, 2018).

[51] Toma, M. The Emerging Use of SPH In Biomedical Applications (2017).

[52] Wang Q, Sun W (2013). Finite element modeling of mitral valve dynamic deformation using patient-specific multi-slices computed tomography scans. Annals of biomedical engineering.

[53] Liang L (2017). Machine learning–based 3‐D geometry reconstruction and modeling of aortic valve deformation using 3‐D computed tomography images. International journal for numerical methods in biomedical engineering.

[54] Icardo JM, Colvee E, Revuelta JM (2013). Structural analysis of chordae tendineae in degenerative disease of the mitral valve. International journal of cardiology.

[55] Pham T (2017). Finite Element Analysis of Patient-Specific Mitral Valve with Mitral Regurgitation. Cardiovascular engineering and technology.

[56] Castillo JG, Anyanwu AC, El-Eshmawi A, Adams DH (2013). All anterior and bileaflet mitral valve prolapses are repairable in the modern era of reconstructive surgery. European Journal of Cardio-Thoracic Surgery.

[57] Hayek E, Gring CN, Griffin BP (2005). Mitral valve prolapse. The Lancet.

[58] Geis, N. *et al*. Percutaneous repair of severe mitral valve regurgitation secondary to chordae rupture in octogenarians using MitraClip. *Journal of interventional cardiology* (2017). 10.1111/joic.12455 29027267

[59] Wang Q, Primiano C, Sun W (2014). Can isolated annular dilatation cause significant ischemic mitral regurgitation? Another look at the causative mechanisms. Journal of biomechanics.

[60] SIMULIA. (ed Manual Dassault Systemes Simulia Corp) (RI: Providence, 2016).

[61] Holzapfel GA, Gasser TC (2001). A viscoelastic model for fiber-reinforced composites at finite strains: Continuum basis, computational aspects and applications. Comput Method Appl M.

[62] Ogden RW (1972). Large Deformation Isotropic Elasticity - On the Correlation of Theory and Experiment for Incompressible Rubberlike Solids. Proceedings of the Royal Society of London. A. Mathematical and Physical Sciences.

[63] Martin C, Sun W (2012). Biomechanical characterization of aortic valve tissue in humans and common animal models. J Biomed Mater Res A.

[64] Pham T, Sun W (2014). Material properties of aged human mitral valve leaflets. J Biomed Mater Res A.

[65] Mokadam NA, Stout KK, Verrier ED (2011). Management of acute regurgitation in left-sided cardiac valves. Texas Heart Institute Journal.

[66] Patel H, Desai M, Tuzcu EM, Griffin B, Kapadia S (2014). Pulmonary hypertension in mitral regurgitation. Journal of the American Heart Association.

[67] Mao W, Li K, Sun W (2016). Fluid–Structure Interaction Study of Transcatheter Aortic Valve Dynamics Using Smoothed Particle Hydrodynamics. Cardiovascular engineering and technology.

