# Author Correction: New insights into mitral heart valve prolapse after chordae rupture through fluid–structure interaction computational modeling

**DOI:** 10.1038/s41598-019-44072-y

**Published:** 2019-06-27

**Authors:** Andrés Caballero, Wenbin Mao, Raymond McKay, Charles Primiano, Sabet Hashim, Wei Sun

**Affiliations:** 1 The Wallace H. Coulter Department of Biomedical Engineering, Georgia Institute of Technology and Emory University, Atlanta, GA USA; 2 Cardiology and Cardiac Surgery, The Hartford Hospital, Hartford, Connecticut USA

Correction to: *Scientific Reports* 10.1038/s41598-018-35555-5, published online 23 November 2018

A supplementary file containing Tables S1 and S2 was omitted from the original version of this Article. This has been corrected in the HTML version of the Article; the PDF version was correct at the time of publication.

