# Supplementary material for: New insights into mitral heart valve prolapse after chordae rupture through fluid–structure interaction computational modeling
Source: Sci Rep. 2018 Nov 23;8:17306. doi: 10.1038/s41598-018-35555-5 (PMC6251907; doi:10.1038/s41598-018-35555-5)
Supplement: Supplementary file 1 — Supplementary Information [file 41598_2018_35555_MOESM1_ESM.docx]

**New insights into mitral heart valve prolapse after chordae rupture through fluid–structure interaction computational modeling**

Andrés Caballero ^1^, Wenbin Mao ^1^, Raymond McKay ^2^, Charles Primiano ^2^, Sabet Hashim ^2^, Wei Sun ^1 *^

*^1.^ The Wallace H. Coulter Department of Biomedical Engineering,*

*Georgia Institute of Technology and Emory University, Atlanta, GA, USA*

*^2.^ Cardiology and Cardiac Surgery, The Hartford Hospital, Hartford, Connecticut, USA*

**Supplementary Information**

**Supplementary Table 1. Summary of chordae and PM forces (N) at peak systole, and percentage change compared to the control model**

|  | Control | Isolated P3 | | Isolated P1 | | Isolated P2 | | Partial P2/P1 | | Partial P2/P3 | | P2/P3 | | P2/P1 | |
| --- | --- | --- | --- | --- | --- | --- | --- | --- | --- | --- | --- | --- | --- | --- | --- |
|  | **(N)** | **(N)** | **(%)** | **(N)** | **(%)** | **(N)** | **(%)** | **(N)** | **(%)** | **(N)** | **(%)** | **(N)** | **(%)** | **(N)** | **(%)** |
| AML marginal | 1.61 | 1.63 | 1.22 | 1.92 | 19.04 | 1.71 | 6.28 | 1.36 | -15.64 | 1.55 | -4.05 | 1.95 | 20.65 | 1.99 | 23.62 |
| AML strut | 3.49 | 3.11 | -11.13 | 3.29 | -5.97 | 3.15 | -9.88 | 3.13 | -10.39 | 2.98 | -14.87 | 2.68 | -23.34 | 2.56 | -26.71 |
| PML marginal | 0.70 | 0.49 | -30.00 | 0.35 | -50.55 | 0.52 | -25.87 | 0.88 | 26.51 | 1.43 | 105.04 | 1.03 | 47.08 | 0.28 | -60.47 |
| PML intermediate | 1.11 | 0.94 | -14.97 | 0.65 | -41.54 | 0.43 | -60.82 | 2.01 | 82.06 | 0.64 | -42.28 | 0.58 | -47.26 | 1.36 | 23.39 |
| PML basal | 4.97 | 4.28 | -13.75 | 4.64 | -6.48 | 4.35 | -12.33 | 2.45 | -50.58 | 2.37 | -52.25 | 1.71 | -65.62 | 1.74 | -65.06 |
| APM | 6.15 | 5.43 | -11.66 | 5.52 | -10.20 | 5.39 | -12.34 | 3.76 | -38.77 | 5.99 | -2.61 | 5.70 | -7.32 | 2.45 | -60.10 |
| PPM | 5.73 | 5.02 | -12.39 | 5.32 | -7.13 | 4.78 | -16.59 | 6.08 | 6.08 | 2.98 | -48.02 | 2.25 | -60.79 | 5.48 | -4.40 |

**Supplementary Table 2. Material parameters of cardiac tissues**

| **MHGO model** | $C_{10}$($\mathrm{kPa}$) | $C_{01}$ | $k_{1}$ ($\mathrm{kPa}$) | $k_{2}$ | $\theta(^{\circ})$ | $\kappa$ | $D (\mathrm{kP}a^{-1})$ |
| --- | --- | --- | --- | --- | --- | --- | --- |
| AV leaflets | 1.738 | 11.368 | 2159.4 | 1158.9 | 4.59 | 0.2359 | 1.0e-5 |
| AML | 0.1245 | 13.665 | 11.007 | 84.84 | 13.09 | 0.0800 | 1.0e-5 |
| PML | 0.0502 | 15.004 | 3.021 | 144.48 | 25.51 | 0.0534 | 1.0e-5 |
| **Ogden model** | $\mu_{1}$($\mathrm{kPa}$) | $a_{1}$ | $\mu_{2}$($\mathrm{kPa}$) | $a_{2}$ | $\mu_{3}$($\mathrm{kPa}$) | $a_{3}$ |  |
| Basal and intermediate chordae | 10256.1 | 16.579 | 10653.8 | 16.554 | 10671.3 | 16.554 |  |
| Strut chordae | 24341.7 | 11.338 | 10331.9 | 11.167 | 14913.6 | 11.188 |  |
| Marginal chordae | 12995.5 | 15.651 | 13082.9 | 15.683 | 12869.7 | 15.662 |  |
